# Delivering Health Education to Children With Chronic Conditions: A Scoping Review and Evidence and Gap Map

**DOI:** 10.1111/cch.70271

**Published:** 2026-04-07

**Authors:** Meredith Smith, Nicole Pope, Nadine Smith, Georgina Henry, Ingrid Honan, Kiara Corso, Grace Evans, Caitlin Doyle, Adrienne Harvey

**Affiliations:** ^1^ School of Allied Health and Human Performance Adelaide University Adelaide South Australia Australia; ^2^ Novita Adelaide South Australia Australia; ^3^ Murdoch Children's Research Institute Melbourne Victoria Australia; ^4^ Monash Centre for Health Research and Implementation, Faculty of Medicine, Nursing and Health Sciences, Monash University, in partnership with Monash Health Melbourne Victoria Australia; ^5^ Child Health Evaluative Services, Research Institute, The Hospital for Sick Children Toronto Ontario Canada; ^6^ Kids Rehab, Perth Children's Hospital Nedlands Western Australia Australia; ^7^ Physiotherapy Department Perth Children's Hospital Nedlands Western Australia Australia; ^8^ Cerebral Palsy Alliance Research Institute, Specialty of Child and Adolescent Health, Sydney Medical School, The University of Sydney Sydney New South Wales Australia; ^9^ Neurodisability and Rehabilitation Murdoch Children's Research Institute Parkville Victoria Australia; ^10^ Melbourne Medical School The University of Melbourne Melbourne Victoria Australia; ^11^ Australian Centre for Precision Health, School of Public Health, Adelaide University Adelaide South Australia Australia; ^12^ South Australian Health and Medical Research Institute (SAHMRI) Adelaide University Adelaide South Australia Australia; ^13^ School of Primary and Allied Health Care, Faculty of Medicine, Nursing and Health Science Monash University Clayton Victoria Australia

**Keywords:** accessibility, children, chronic conditions, health education

## Abstract

**Introduction:**

Developmentally appropriate health education is essential for children with chronic conditions and their families to support self‐management and improve quality of life. Although a range of educational approaches have been reported, no comprehensive review has examined how these interventions are developed, which approaches are most used, or their outcomes. Accessibility is also critical given diverse cognitive, communication and motor abilities within paediatric populations. This review aimed to map and synthesise evidence on health education interventions for children (5–12 years) with chronic conditions.

**Methods:**

Studies reporting health education interventions for children (5–12 years) with chronic conditions were identified from MEDLINE, CINAHL, PsycINFO, ERIC, Scopus and Cochrane Library. Data were extracted using predefined categories, including delivery approaches aligned with Saxby's (2020) framework for paediatric supported self‐management. Participant (knowledge, behaviour change or health/symptom) and implementation outcomes were extracted. Findings were synthesised using an Evidence and Gap Map to identify strengths and gaps in delivery and implementation.

**Results:**

A total of 118 studies were included. Most interventions targeted endocrine/metabolic conditions and were delivered in hospital outpatient settings. Health professionals primarily facilitated education, though self‐directed and digital facilitators are emerging. Delivery was mainly face‐to‐face, with increasing use of remote approaches. Over half of studies incorporated multiple recommended approaches: peer/cooperative learning (50%), story/play‐based strategies (35%), caregiver involvement (27%), pictorial representation (25%) and active/experiential learning (14%). Most studies reported at least one participant outcome (86%) and nearly three‐quarters reported an implementation outcome (73%). Only 14% of studies addressed accessibility considerations, predominantly for children with diverse cognitive abilities.

**Conclusions:**

This review highlights a growing emphasis on active learning strategies to support health education for chronic conditions, moving beyond passive learning. To improve inclusivity and accessibility, interventions should prioritise individualised content, multimodal delivery and flexible approaches with meaningful involvement of children and families in intervention design and development.

## Summary

1. There is a growing emphasis on active learning strategies to support health education for paediatric chronic conditions.
2. Health education interventions should be designed to be inclusive and accessible for children with diverse cognitive, communication and movement abilities.
3. Individualised content, multimodal delivery and flexible approaches can be used to improve accessibility of health education interventions.
4. Children and families with diverse abilities should be meaningfully involved in health education intervention design.

## 1 Introduction

Chronic health conditions, defined by the Australian Institute of Health and Welfare as ‘complex, long‐lasting conditions with ongoing effects, including social and economic consequences,’ are common in paediatric populations (Australian Institute of Health and Welfare 2024), with an estimated prevalence of up to 30% among children and adolescents (Wisk and Sharma 2025). These conditions include noncommunicable diseases and permanent disabilities (e.g., cerebral palsy and spinal cord injury) and can significantly impact quality of life (Silva et al. 2019) and may have adverse long‐term outcomes (Mastorci et al. 2025) (e.g., emotional wellbeing, impact on participation in daily routines). Management of paediatric chronic health conditions is most successful when medical management is combined with health education, delivered through collaborative partnerships between the patient, family and care providers (Lozano and Houtrow 2018).

The World Health Organization broadly defines health education as any learning experience involving communication aimed at improving knowledge and developing life skills related to individual and/or community health (World Health Organization 2012). In the context of chronic health conditions, this includes self‐management education, which focuses on supporting patients to develop problem‐solving and decision‐making skills to achieve the best possible quality of life with their condition (Bodenheimer et al. 2002). For children, this is more appropriately referred to as ‘supported self‐management,’ recognising children receive health care in the context of their family and broader support networks. The child's ability to engage with health education, and to independently implement self‐management strategies, progressively increases with developmental maturity (Lozano and Houtrow 2018).

Across the literature, the effectiveness of supported self‐management has been primarily measured via symptom and clinical outcomes. However, recent consensus guidelines on developmentally appropriate supported self‐management for children and young people with chronic conditions emphasise knowledge acquisition, behaviour change and implementation of health education as foundational mechanisms underpinning longer‐term health outcomes (Saxby et al. 2020). These guidelines articulate overarching principles and age‐specific recommendations relating to knowledge, involvement, care planning, monitoring and responding, transitions, social impacts, healthy lifestyle behaviours and access to support services (Saxby et al. 2020). Accordingly, understanding how health education interventions function, for whom, and under what conditions requires evaluating outcomes beyond symptoms and clinical measures, including knowledge, behaviour change and implementation related domains.

Understanding how to best deliver health education in alignment with these recommendations is crucial when designing effective interventions. This includes consideration of the method of delivery (i.e., digital platforms, face to face interactions, video‐based approaches, storytelling, peer‐led initiatives and group‐based interventions), the setting, facilitator, content and level of support (i.e., self‐guided through to real‐time health professional interactions) (Rivero‐Santana et al. 2021). While some research has explored health education delivery methods for adolescents with chronic health conditions (Gauci et al. 2022), understanding the range and suitability of approaches available for younger children, particularly in relation to supported self‐management recommendations, is limited.

Many children with chronic health conditions also have diverse cognitive, communication and movement abilities, and this presents unique challenges when designing appropriate health education interventions. For example, cerebral palsy is the most common childhood onset physical disability, which affects movement and posture and is often accompanied by additional co‐occurring conditions. It is estimated that one in two children with cerebral palsy have an intellectual disability (Novak et al. 2012), and the Australian Cerebral Palsy Register estimates 38% of children with cerebral palsy have some form of communication impairment at 5 years of age (Australian Cerebral Palsy Register 2023). Furthermore, these children often experience multiple co‐occurring chronic health conditions requiring ongoing management, such as chronic pain and epilepsy. Children with chronic health conditions and diverse cognitive, communication and movement abilities, therefore, require health education that is both accessible and developmentally appropriate to support effective self‐management.

The overarching aim of this scoping review was to explore and map existing evidence on health education interventions for children aged 5–12 years with chronic health conditions across a range of settings, while also considering the needs of children with diverse cognitive, communication and movement abilities. A preliminary search of MEDLINE, the Cochrane Database of Systematic Reviews and *JBI Evidence Synthesis* was conducted and no current or underway systematic or scoping reviews addressing this topic were identified. The following research questions were used to guide the review:

1. What is known about how health education is delivered to paediatric populations aged 5–12 years with chronic health conditions?
2. What participant (e.g., knowledge, behaviour change and health/symptom) and implementation outcomes have been examined in studies of health education interventions for these populations?
3. To what extent have studies considered inclusion and accessibility for children aged 5–12 years with chronic health conditions, including those with diverse cognitive, communication and movement abilities?

## 2 Methods

### 2.1 Protocol and Registration

The protocol for this review was guided by the Joanna Briggs Institute Manual for Evidence Synthesis for Scoping Reviews (Peters et al. 2020). The results were then synthesised into an Evidence and Gap Map (EGM), which is a web‐based visual tool to display available evidence for a research question (White et al. 2020; Campbell et al. 2023). The PRISMA Extension for Scoping Reviews Guidelines were followed for quality reporting (*Annals of Internal Medicine* 2018). The review protocol was preregistered and published on Open Science Framework (https://osf.io/9zxeq).

### 2.2 Eligibility Criteria

Inclusion and exclusion criteria were defined using the PCC (population, concept and context) criteria (Table 1) (Peters et al. 2020). Studies available in any language from the past 10 years (2015–2025) were included if they (1) represented children aged 5–12 years (or studies where data for this age group could be extracted separately) with chronic health conditions (Pope et al. 2025) (e.g., cancer, diabetes, brain‐based disabilities, chronic pain, respiratory conditions), (2) described a health education intervention on any health‐related topic delivered using any method, (3) were a primary study of any type with the purpose of evaluating the health education intervention (e.g., randomised controlled trial, qualitative study and mixed methods studies) and (4) reported a participant or implementation outcome of the health education. Participant outcomes were knowledge, behaviour change and symptom/health outcomes, however symptom/health outcomes were only reported if they were presented alongside knowledge/behaviour change or implementation outcomes. Implementation outcomes were defined according to Proctor's Framework, an approach that helps researchers and practitioners evaluate how well an evidence‐based intervention is being implemented. This framework outlines eight conceptually distinct implementation outcomes: acceptability, adoption, appropriateness, feasibility, fidelity, implementation cost, penetration and sustainability (Proctor et al. 2011). Excluded were (1) studies that described the education intervention without any evaluation, (2) studies in the general paediatric population, (3) health education interventions delivered solely to parents/caregivers or healthcare providers without direct child engagement or studies that only reported parent or healthcare provider outcomes, (4) studies only reporting the outcomes of the health condition itself (i.e., severity), without any other participant/implementation outcomes and (5) systematic reviews to avoid duplication of evidence and to maintain a clear focus on the review's objectives.

**TABLE 1:** Inclusion and exclusion criteria for study screening.

Criteria	Include	Exclude
Population	Children aged 5–12 years (or studies where data for this age group can be extracted separately) with a chronic health condition Chronic health conditions include cancer, diabetes, brain‐based disabilities, chronic pain, gastrointestinal conditions and neurological conditions	Studies including the general paediatric population, i.e., the general paediatric population receiving education on prevention of chronic health conditions, i.e., obesity, healthy eating Studies focused exclusively on children under 5 or over 12 years, or with no extractable subgroup data
Concept	Health education on any health‐related topic (e.g., disease management, prevention, health promotion and self‐management education) Delivered using any method (e.g., digital, face‐to‐face, group‐based, storytelling, visual aids, videos)	Health education delivered solely to parents/caregivers or healthcare providers without direct child engagement Studies that report only parent outcomes or healthcare provider outcomes Studies only reporting outcomes of the health condition itself (i.e., severity)
Context	Diverse settings including hospital, school, home, digital, online	
Types of evidence source	Quantitative and qualitative published articles	Grey literature Systematic reviews (after citation searching for relevant articles)

### 2.3 Search Strategy

The search strategy was developed with the research team and a health sciences librarian. The databases searched were MEDLINE (via Ovid), CINAHL, PsycINFO, ERIC, Scopus and Cochrane Library on 30th April 2025. The database search strings are available in Data S1. Additional records were identified by citation searching the reference lists of included studies.

#### 2.3.1 Study Screening

All identified citations were collated and uploaded into EndNote 21 (Clarivate Analytics, PA, USA) and duplicates removed. These were then imported into Covidence (Covidence systematic review software, Veritas Health Innovation, Melbourne, Australia. Available at www.covidence.org). Following pilot testing, 13 independent reviewers (M.S., N.S., G.E., S.S., C.D., K.C., J.H., A.K., N.P., K.B., C.B., H.G. and I.H.) were involved in title and abstract screening; each study was screened by two people. Nine authors completed full text screening (M.S., N.S., G.E., N.P., K.C., G.H., C.D., K.B. and I.H.) with at least two reviewing each abstract and full text. Where conflicts occurred, these were resolved through consensus with another author (A.H.). Training sessions took place prior to the beginning of the screening, with researchers confirming their preparedness through discussion during these sessions. Regular consensus meetings were then held throughout the screening phase to ensure consistent application of the eligibility criteria and to resolve any concerns.

#### 2.3.2 Data Extraction

Eight authors (M.S., N.S., G.E., N.P., K.C., G.H., C.D. and I.H.) were involved in data extraction using a custom data extraction tool developed by the project team. Pilot testing of six studies was conducted prior to commencing the data extraction phase to refine the data extraction tool and criteria, with regular consensus meetings held to address discrepancies, refine extraction criteria and ensure a consistent approach across all reviewers. For each included record, data extraction was completed by one author from the team of eight, with a second author independently checking a random 20% sample of records. Authors completed all data extraction manually. After manual extraction was finalised, AI‐assisted extraction outputs generated by Elicit (https://elicit.com) were made available to authors as an optional crosscheck. No AI extracted data were used in the analysis or reported in the review. Agreement between manual and AI generated extraction was not formally assessed, although authors noted that the AI outputs were generally helpful but occasionally inaccurate or incomplete. The following data were extracted from the included studies: (1) study characteristics (i.e., author, publication year, funding, country of study, study design and study population), (2) information on the health education intervention delivery and content (mapped to the supported self‐management consensus recommendations (Saxby et al. 2020)) and (3) health education intervention outcomes including both participant outcomes (knowledge, behaviour change and health/symptom outcome) and implementation outcomes (mapped to Proctor's implementation outcomes (Proctor et al. 2011)).

All records were independently evaluated for quality using the Mixed Methods Appraisal Tool (MMAT‐v2018) (Hong et al. 2018; Pace et al. 2012) as it is applicable across various study types. Although it is not usual practice to include quality appraisal in a scoping review, we included the MMAT to allow the evidence to be included in the EGM. We used the adapted MMAT scoring system described by Pope et al. (2025) to categorise the quality of the scientific evidence for the EGM as high, moderate, low or critically low. Studies were rated as high quality if they met 4–5 of the methodological quality criteria specified for their respective study type (with ‘yes’ responses), moderate if they met 2–3 criteria, low if they met 1 criterion and critically low if they met none. A study was deemed to not meet a criterion if reported as not meeting the requirements, or if there was insufficient information to evaluate the criterion (‘cannot tell’ response).

## 3 Results

**FIGURE 1:**
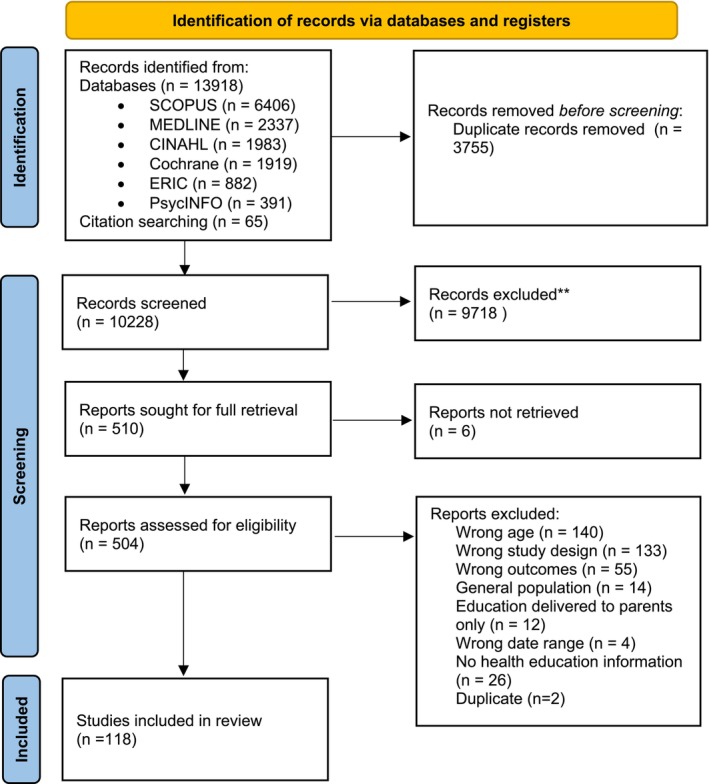
PRISMA 2020 flow diagram of study identification and screening.

Database searches identified 13 918 records. An additional 65 records were found through citation searching. After removing 3755 duplicates, 10 228 records were screened against the inclusion and exclusion criteria (see Table 1), and 504 full‐text articles were assessed for eligibility. Ultimately, 118 studies were included in the review. Reasons for exclusion are shown in the PRISMA flow diagram (*Annals of Internal Medicine* 2018) in Figure 1. A full list of included studies is provided in Data S2 and included in the interactive EGM (https://healtheducationegm.netlify.app/ or https://doi.org/10.25909/31101373). Each record in the EGM contains its title, authorship, publication year, scientific publication details and URL (where possible).

### 3.1 Study Characteristics and Overall Quality of Evidence

Of the 118 studies included, the most common design was randomised controlled trials (RCTs) (*n* = 42, 35.6%), followed by nonrandomised experimental studies (*n* = 38, 32.2%) and qualitative research (*n* = 12, 10.2%). Most studies were conducted in the United States of America (*n* = 40, 33.9%). Quality ratings using MMAT indicated that nearly all studies were either ‘high’ (*n* = 54, 45.8%) or ‘moderate’ (*n* = 54, 45.8%) quality. Individual study quality ratings are available in the EGM.

### 3.2 Participants and Population

Across all included studies (*n* = 118), there were 8951 participants. Most studies included participants across the 5‐ to 12‐year age range (*n* = 109, 92.3%), with older children more commonly represented (8–10 years: *n* = 109; 11–12 years: *n* = 98) compared to younger children (5–7 years: *n* = 75). The chronic health conditions studied were respiratory (*n* = 20 studies, 4182 participants), endocrine/metabolic (*n* = 36 studies, 1257 participants), oncological (*n* = 16 studies, 878 participants), neurodevelopmental (*n* = 21 studies, 521 participants), haematological (*n* = 4 studies, 254 participants), mental health (*n* = 1 study, 134 participants), neurological (*n* = 3 studies, 55 participants), obesity/weight‐related (*n* = 6 studies, 1205 participants), rheumatological (*n* = 4 studies, 337 participants) and other (*n* = 6 studies, 216 participants). Five studies included multiple condition categories. Within the neurodevelopmental category, studies included participants with autism (*n* = 12), spina bifida (*n* = 1), attention deficit hyperactivity disorder (*n* = 4), intellectual disability (*n* = 3) and cerebral palsy (*n* = 1).

### 3.3 Health Education Delivery

#### 3.3.1 Settings and Facilitators

Health education was most often delivered in hospital outpatient settings (*n* = 51, 43.2%), followed by the home (*n* = 28, 23.7%) and online/virtual environments (*n* = 22, 18.6%). Twenty‐five studies provided education across multiple settings, most commonly home and online or home and hospital. Delivery was primarily by health professionals (*n* = 64, 54.2%), including allied health, medical practitioners and nurses. Self‐directed education was also common (*n* = 29, 24.6%), followed by parent/caregiver‐led (*n* = 18, 15.3%) and digital agent‐led (*n* = 18, 15.3%). Nearly half (*n* = 50, 42.7%) of the studies used a combination of facilitators, most often a mix of healthcare professionals, self‐directed plus digital agent or self‐directed plus parent/caregiver. Digital agents included avatars or virtual characters within augmented reality, mobile apps and digital games, while robots were used in face‐to‐face interactions.

#### 3.3.2 Methods of Delivery

Health education was most often delivered individually (*n* = 61, 52.7%) or in groups (*n* = 50, 42.4%), with a small number using both formats (*n* = 7, 5.9%). Most sessions were face‐to‐face (*n* = 69, 58.5%), while just over one‐third were remote (*n* = 42, 35.6%)—typically self‐paced or asynchronous. When assessed against the supported self‐management health education recommendations for 5‐ to 12‐year‐olds with chronic health conditions (Saxby et al. 2020), over half of the studies (*n* = 75, 63.6%) used more than one recommended approach. Common methods included peer or cooperative learning (*n* = 59, 50.0%), story‐based/play‐based strategies (*n* = 41, 34.7%), caregiver involvement (*n* = 32, 27.1%), pictorial representation (*n* = 30, 25.4%) and active/experiential learning (*n* = 16, 13.6%). Only four studies did not include any recommended methods. Technology‐assisted learning was used in 45 studies (38.1%). Examples of specific activities aligned with these methods are provided in Table 2.

**TABLE 2:** Delivery methods and activities mapped to the supported self‐management of chronic conditions consensus education delivery recommendations.

Supported self‐management of chronic conditions for 5‐ to 12‐year‐olds consensus recommendation	Delivery methods used to meet this recommendation	Example of a specific activity from an included article
Active/experiential learning	Augmented reality* Virtual reality* Robotics* Hands‐on practical	3‐ to 8‐year‐olds with Autism used an augmented reality app (via a tablet) which had a virtual character guiding the child through a visual schedule of bedtime routines to improve sleep and sleep routine independence (Ahmadian et al. 2025).
Caregiver supported learning	Robotics* Discussion‐based workshops Camps	A robot delivered sleep‐hygiene education to children aged 8–12 years with cancer and their parents. This involved social interaction with the robot and quiz activities (van Bindsbergen et al. 2022).
Peer/cooperative learning	Discussion‐based workshops Digital applications (mobile apps, websites) * Robotics* Camps Coaching/counselling	A social story used to deliver sexual education to people with Autism aged 11–15 years (Stankova and Trajkovski 2021). A residential camp for children aged 6–17 years with type 1 diabetes mellitus to learn knowledge about diabetes self‐management and practice insulin injection and monitoring (Hill et al. 2015).
Story‐based or play‐based learning	Games (video games*, board games) Role play/simulation Storytelling (including social stories) Books (comic books, picture books) Puppet shows	A serious video game for 7–12‐year‐olds with type 1 diabetes mellitus called ‘Heroes of Diabetes—The Power of Knowledge’. The main character in the game has diabetes, and the child performs missions to learn more about their illness and self‐care tasks (Sparapani et al. 2023).
Pictorial representation	Videos* Printed materials (booklets, colouring books, comic strips)	A comic strip for children aged 5–13 years with type 1 diabetes mellitus to help them understand the function of insulin, warning signs of hypo/hyperglycaemia and the use of insulin (da Silva et al. 2023).

* Indicates a technology‐assisted learning method.

While the supported self‐management health education recommendations (Saxby et al. 2020) were consistently applied across age groups, the methods used varied. Younger children typically engaged with puppets or simple picture books for story/play‐based education, whereas older children were offered more complex activities such as serious video games or board games. Caregiver‐supported learning was present across all age groups, but for younger children (5–7 years) this was more hands‐on (e.g., participating in sessions), compared to an assisting role for older children (e.g., setting up augmented reality or supporting home‐based implementation). Children with medical conditions (e.g., type 1 diabetes mellitus) often used discussion‐based activities for peer/cooperative learning, while those with neurodevelopmental conditions relied more on pictorial representation within peer learning environments, almost always with caregivers present.

#### 3.3.3 Frequency of Sessions and Resources Required

Most education was delivered in a sequenced format, involving more than one interaction (*n* = 73, 62%). These were typically short series (2–4 sessions, *n* = 26, 35.6%), medium series (5–8 sessions, *n* = 28, 38.4%) or long/multiweek programs (*n* = 19, 26.0%). Long/multiweek programs typically ran for 8–14 weeks, with engagement ranging from daily activities to 1–3 sessions per week. The session lengths varied widely (10‐ to 20‐min activities to longer 1 to 2‐h structured sessions), resulting in total weekly involvement of approximately 1–4 hours. Resources were required in nearly all studies (*n* = 115, 97.5%), although costs were rarely reported. Technology‐assisted learning often required access to a device (usually a tablet) and an internet connection. Some activities, such as residential camps, were highly resource‐intensive, requiring campsites, large facilities and significant numbers of volunteers or staff.

#### 3.3.4 Content and Co‐Design Involvement

Educational content varied widely, but most studies focused on disease‐specific knowledge (*n* = 93, 78.8%), followed by self‐monitoring and daily management (*n* = 48, 40.7%), treatment understanding (*n* = 45, 38.1%) and coping or behavioural skills (*n* = 40, 33.9%). Only nine studies explicitly included individualised content. Co‐design was incorporated in just over one‐third of studies (*n* = 39, 33%), most often with clinicians (*n* = 26) and not the patient population, although descriptions of their roles in the co‐design process were generally limited. Eighteen studies involved multiple stakeholders, such as children, parents/caregivers and clinicians, in the co‐design process.

### 3.4 Health Education Outcomes

Health education outcomes were reported as participant outcomes (knowledge, behaviour change and symptom/health outcomes) and Proctor's implementation outcomes (Proctor et al. 2011). Most studies reported at least one participant outcome (*n* = 101, 85.5%), with nearly half reporting knowledge outcomes (*n* = 58, 49.2%). Almost three‐quarters of studies reported at least one implementation outcome (*n* = 86, 72.9%), most commonly acceptability (*n* = 64) and feasibility (*n* = 45). No studies assessed sustainability, and only four reported on cost or penetration. Outcome measurement was primarily through study‐specific tools, such as surveys, questionnaires or interviews, with minimal consistency across studies.

### 3.5 Accessibility Considerations

Sixteen of the included studies (13.6%) explicitly reported content or methods designed to be inclusive of people with diverse accessibility needs (Table 3). All were studies conducted in populations with known disabilities (i.e., autism, hearing impairment, spina bifida, etc.). The majority of these were adaptations for those with diverse cognitive abilities (*n* = 11), with only a few studies adapting for communication (*n* = 3), hearing (*n* = 2), movement (*n* = 2) or vision (*n* = 1) impairment. Four studies considered other accessibility considerations such as sensory and cultural needs.

**TABLE 3:** Summary of adaptations made for accessibility needs in the included studies.

Accessibility area	Summary of adaptations made
Cognition	Personalisation and customisation of educational content, languages, tasks and characters to reflect the child's preferences Simplified language and minimal text Support from an adult to read educational stories Multiple learning strategies (i.e., combination of video, audio and hands‐only learning tasks) engaged to accommodate different learning styles Visual aids to support learning Predictable structures to the learning program Play‐based content Hands‐on learning activities such as cooking, role‐plays, arts activities, dolls/models Smaller groups or individual sessions Flexible teaching methods if the child is not understanding (i.e., providing guidance through gestures, verbal or physical guidance)
Communication	1:1 support from an adult to read and engage with educational stories Visuals as a form of augmentative and alternative communication
Hearing	Closed caption on videos Visual aids to support learning Sign language options
Movement	Inclusive movement options for physical activity (i.e., wheelchair‐based activities)
Vision	Narration on videos
Other	Provided activities to manage sensory overstimulation. Gentle educational approach to accommodate anxiety and overstimulation Providing educational content in multiple languages

Accessibility is particularly important for the condition category of neurodevelopmental conditions, given the known diversity of cognitive, communication and movement abilities within these populations. Within this condition category, most studies focused on children with autism (*n* = 12, 57%), followed by attention‐deficit/hyperactivity disorder (ADHD) (*n* = 3, 14%) and intellectual disability (*n* = 3, 14%). Despite this, four studies (Slater et al. 2024; Pingul et al. 2017; Bul et al. 2016; Alves Kaneto et al. 2018) conducted in neurodevelopmental populations explicitly excluded participants with cognitive impairment or intellectual disability.

The delivery methods used in populations of people with known disabilities were not substantially different from those used in other populations; however, the education methods tended to require a shorter attention span (i.e., short video clips, < 1 min), have greater caregiver involvement, providing hard copy visuals for use in both the educational setting and at home, and have storytelling with an adult facilitating rather than independent reading.

## 4 Discussion

This scoping review explored and mapped existing evidence on health education interventions for children aged 5–12 years with chronic health conditions, with specific consideration of children with diverse cognitive, communication and movement abilities. Most studies focused on endocrine/metabolic conditions and were delivered in hospital outpatient settings. While health professionals primarily facilitated the health education interventions, self‐directed education and digital agent facilitators were emerging methods. Similarly, although most health education was delivered in face‐to‐face settings, the use of remote methods is increasing. Pleasingly, over half of the studies included more than one of the recommended delivery approaches for supported self‐management education (Saxby et al. 2020). Most studies reported at least one participant outcome (knowledge, behaviour change or symptom/health outcome) and nearly three‐quarters reported at least one implementation outcome. However, accessibility needs were explicitly addressed in only 14% of studies, primarily for children with diverse cognitive abilities.

### 4.1 Health Education Components and Outcomes

More than half of the included studies utilised more than one recommended approach for self‐management health education, including peer or cooperative learning. While peer involvement commonly occurred through cooperative group‐based learning, only three studies (Armbrust et al. 2015; Hughes et al. 2015; Sullivan‐Bolyai et al. 2016) used a peer‐led education delivery approach. Given the growing evidence supporting the benefits of peer‐led learning for school‐aged children, this presents a notable opportunity for future intervention development (Wade et al. 2022). Furthermore, with the emerging increase in digital agent‐led health education seen in this review, digital or virtual peers may represent an evolving form of ‘peer’ education, offering opportunities for greater customisation and individualisation. This may be particularly valuable for children with limited opportunities to meet peers living with similar conditions or unique learning or support needs.

Caregivers were involved in just over one quarter of the included studies. Given that two key supported self‐management recommendations for this age group explicitly involve caregivers, ‘encouraging caregivers to give the child increasing responsibility in communicating with clinicians’ and ‘encouraging caregivers to model daily self‐care and health activities’ (Saxby et al. 2020), this presents an important area for further consideration and improvement. Health education interventions that adopt a family‐based approach, rather than targeting children or caregivers separately, may be better positioned to operationalise these recommendations. Evidence from health education interventions outside the chronic condition population suggests that involving children and caregivers together promotes a more unified family approach to intervention participation and behaviour change (Parekh et al. 2020).

Although nearly three‐quarters of studies reported at least one implementation outcome, only four studies (Pingul et al. 2017; Armbrust et al. 2015; Pemberton et al. 2022; Robertson et al. 2017) included information on the cost of the health education intervention. This represents a significant limitation because cost data are critical across all stages of implementation, including feasibility, adoption, scalability and long‐term sustainability. Without cost reporting, it is difficult to assess whether interventions can be transferred from research settings into routine practice (Proctor et al. 2011). Future health education interventions should include cost‐related outcomes to improve the assessment of real‐world feasibility and implementation potential.

### 4.2 Accessibility of Health Education Interventions

While it was encouraging that some health education interventions reported strategies designed to be inclusive of children with diverse needs, over half of these studies were specifically focused on autism. In many cases, the primary focus was on sensory accessibility, with three autism‐focused studies explicitly excluding children with other needs such as intellectual disability (Slater et al. 2024; Bul et al. 2016; Kral et al. 2023), hearing or visual impairment (Slater et al. 2024; Kral et al. 2023) or physical disabilities (Bul et al. 2016). This highlights a critical gap in the literature and a priority for future research to ensure that the full range of accessibility needs experienced by children with chronic health conditions are adequately addressed. Despite limited explicit reporting of accessibility considerations, several delivery methods used in the nondisability populations may be appropriate for children with diverse needs, with suitable adaptations (see Table 2 for examples). A key principle is enabling individualisation and customisation of content, allowing health education to be tailored to the needs of the individual learner. This approach aligns closely with the supported self‐management consensus recommendations (Saxby et al. 2020). However, in this review, less than 10% of the included studies explicitly reported individualised content. Furthermore, only 22% (*n* = 26) involved children or their caregivers in the development of the health education intervention.

Future health education interventions should therefore prioritise greater individualisation and co‐design, which are likely key to improving accessibility for children with diverse needs. In addition, drawing on established accessibility guidelines provides a practical starting point for intervention development. One example is the United Nations Disability Inclusive Communication Guidelines (United Nations 2021), which outline recommendations for accessible content, including multimodal information, accessible imagery and writing, captions/transcripts, audio descriptions and sounds, websites/social media and considerations for in‐person/digital education. While these guidelines are not specific to children, they offer a useful foundation that can be combined with meaningful co‐design involving children and caregivers to improve the relevance, accessibility and usability of future health education interventions.

### 4.3 Strengths and Weaknesses

This review has several strengths, including the mapping of health education interventions to an existing consensus statement on supported self‐management for children with chronic health conditions. The review also employed a rigorous and comprehensive search and screening process, incorporating a novel approach that cross‐checked human data extraction with artificial intelligence‐generated data extraction to enhance accuracy and completeness. Furthermore, this review identified a broad range of participant and implementation outcomes and provided unique insights into how health education interventions have been adapted for children with diverse abilities, informing future intervention development.

Limitations of this review should be acknowledged. Some included studies only provided a brief description of the educational method used, making it difficult to determine whether specific strategies, such as active/experiential learning, were used. To ensure consistency, we defined active/experiential learning as *opportunities for participants to engage in authentic, hands‐on experiences* (Kolb 1984). Some studies may have used this approach but were not identified due to limited descriptive detail. Furthermore, this review did not explicitly address equity‐related characteristics beyond the accessibility requirements for children with diverse abilities. Future reviews could extend this work by examining broader equity considerations, such as interventions designed for regional or remote communities, or culturally and linguistically diverse populations.

## 5 Conclusions

This scoping review examined health education interventions for children aged 5–12 years with chronic health conditions. Although many reported educational methods and outcomes have potential for adaptation for children with diverse needs, very few studies explicitly considered accessibility requirements. Future research should prioritise meaningful engagement with children and parents and use individualised, multimodal and flexible delivery approaches to ensure interventions are inclusive and accessible. Additionally, improved reporting on key implementation outcomes, such as cost, will strengthen understanding of scalability and generalisability. Emerging trends, including self‐directed education and digital facilitators, warrant further exploration to assess effectiveness and accessibility for paediatric populations.

## Author Contributions

M.S., N.P., N.S., G.H., I.H., K.C. and A.H. contributed to study conceptualization and methodology. All authors contributed to data collection and extraction. M.S., N.P. and A.H. contributed to formal analysis. M.S. wrote the original draft, and all authors provided critical review of the final manuscript.

## Funding

This work was supported by the University of Adelaide through a Faculty of Health and Medical Sciences Early Career Grant.

## Supporting information


**Data S1:** Search strategies.

**Data S2:** Full list of included references (https://healtheducationegm.netlify.app/ or https://doi.org/10.25909/31101373).

## Data Availability

The authors confirm that the data supporting the findings of this study are available within the article and its supplementary materials.
