# Supplementary material for: Delivering Health Education to Children With Chronic Conditions: A Scoping Review and Evidence and Gap Map
Source: Child Care Health Dev. 2026 Apr 7;52(3):e70271. doi: 10.1111/cch.70271 (PMC13056421; doi:10.1111/cch.70271)
Supplement: Supplementary file 1 — Data S1: Search strategies. [file CCH-52-e70271-s001.docx]

REVIEW 1 (5-12years) - Medline, CINAHL, PsychINFO, ERIC, Scopus, Cochrane Library

**BASIC SEARCH STRING**

(child* OR pediatric* OR paediatric* OR youth)

AND

(“chronic pain*” OR “widespread chronic pain*” OR “chronic primary pain*” OR “chronic secondary pain*” OR “persistent pain*” OR “ongoing pain” OR “long-term pain*” OR “chronic disease” OR “chronic condition*” OR “chronic illness” OR “long-term illness” OR “disability” OR “Cancer” OR “Diabetes” OR “Autism” OR “cerebral palsy” OR “intellectual disability*” OR “Attention deficit hyperactivity disorder” OR “brain injury” OR “mental health” OR “idiopathic arthritis” or “irritable bowel disease”).

AND

(“health education” OR “community education” OR “community education program*” OR “community health” OR “community health program*” OR “self-manag*” OR “self-management program*” OR “health promotion” OR “disease education” OR “chronic disease education” OR “education* program*” OR “education* resource*” OR “patient education”)

AND

("impact" OR "evaluation" OR "program evaluation" OR "effectiveness” OR "feasibility" OR "acceptability" OR "usability" OR "implementation" OR "adoption" OR "barrier" OR "facilitator" OR "knowledge" OR "health knowledge" OR "behavior change" OR "behaviour change" OR "self-efficacy" OR "health behavior" OR "attitude change" OR "adherence" OR “delivery”)

Limit to ‘school aged’ (5-12 years)

Limit to past 10 years (2015-2025)

**MEDLINE (Ovid) - returning 2237 (30/4/25) (2015-2025)**

Can filter 6-12 years (“child” filter)

(pediatrics.sh. OR exp child/ OR (pe$diatric* OR child* OR youth*).ti,ab.kw )

AND

(chronic pain.sh. OR exp chronic disease/ OR (chronic pain* OR widespread chronic pain* OR chronic primary pain* OR chronic secondary pain* OR persistent pain* OR ongoing pain OR long-term pain* or musculoskeletal pain OR chronic disease OR chronic condition* OR chronic illness OR long-term illness OR disability OR Cancer OR Diabetes OR Autism OR cerebral palsy OR intellectual disability* OR Attention deficit hyperactivity disorder OR brain injury OR mental health OR idiopathic arthritis OR irritable bowel disease).ti,ab,kw..)

AND

(health education.sh. OR self-management.sh. OR health promotion.sh. OR patient education as topic.sh. OR (health education OR community education OR community education program* OR community health OR community health program* OR self-manag* OR self-management program* OR management OR education* program* OR education* resource* OR patient education).ti,ab,kw..)

AND

(health knowledge, attitudes, practice.sh. OR treatment adherence and compliance.sh. OR health education.sh. OR program evaluation.sh. OR exp program evaluation/ OR exp feasibility studies/ OR (impact OR evaluation OR intervention* OR program evaluation OR effective OR feasibility OR acceptability OR usability OR implementation OR adoption OR knowledge* OR health knowledge OR understanding OR behaviour change OR self-efficacy OR adherence OR delivery).ti,ab,kw..)

FROM MEDLINE:

| 1. | (pediatrics.sh. or exp child/ or (pe$diatric* or child* or youth*).ti,ab,kw.) and (chronic pain.sh. or exp chronic disease/ or (chronic pain* or widespread chronic pain* or chronic primary pain* or chronic secondary pain* or persistent pain* or ongoing pain or long-term pain* or musculoskeletal pain or chronic disease or chronic condition* or chronic illness or long-term illness or disability or Cancer or Diabetes or Autism or cerebral palsy or intellectual disability* or Attention deficit hyperactivity disorder or brain injury or mental health or idiopathic arthritis or irritable bowel disease).ti,ab,kw.) and ((health education or self-management or health promotion or patient education as topic).sh. or (health education or community education or community education program* or community health or community health program* or self-manag* or self-management program* or education* program* or education* resource* or patient education or health literacy).ti,ab,kw.) and (health knowledge, attitudes, practice/ or "treatment adherence and compliance"/ or health education.sh. or program evaluation.sh. or exp program evaluation/ or exp feasibility studies/ or (impact or evaluation or program evaluation or effective or feasibility or acceptability or usability or implementation or adoption or knowledge* or health knowledge or behaviour change or self-efficacy or adherence or delivery).ti,ab,kw.) |
| --- | --- |
| 2. | limit 1 to (yr="2015 - 2025" and "child (6 to 12 years)") |

**CINAHL - returning 1983 (30/4/25) (2015-2025)**

Can filter search “child” = 6-12

(

MH "Pediatrics+" OR MH "Child+" OR

TI (pediatric* OR paediatric* OR child* OR youth*) OR

AB (pediatric* OR paediatric* OR child* OR youth*)

)

AND

(

MH "Chronic Pain+" OR

TI (chronic pain* OR "widespread chronic pain*" OR "chronic primary pain*" OR "chronic secondary pain*" OR persistent pain* OR ongoing OR "long-term pain*" OR “chronic disease” OR “chronic condition*” OR “chronic illness” OR “long-term illness” OR “disability” OR “Cancer” OR “Diabetes” OR “Autism” OR “cerebral palsy” OR “intellectual disability*” OR “Attention deficit hyperactivity disorder” OR “brain injury” OR “mental health” OR ‘idiopathic arthritis’ or “irritable bowel disease”) OR

AB (chronic pain* OR "widespread chronic pain*" OR "chronic primary pain*" OR "chronic secondary pain*" OR persistent pain* OR ongoing OR "long-term pain*" OR “chronic disease” OR “chronic condition*” OR “chronic illness” OR “long-term illness” OR “disability” OR “Cancer” OR “Diabetes” OR “Autism” OR “cerebral palsy” OR “intellectual disability*” OR “Attention deficit hyperactivity disorder” OR “brain injury” OR “mental health” OR ‘idiopathic arthritis’ or “irritable bowel disease”)

)

AND

(

MH "Health Education+" OR MH "Self Care+" OR MH "Health Promotion+" OR MH "Patient Education as Topic+" OR

TI (“health education” OR “community education” OR “community education program*” OR “community health” OR “community health program*” OR “self-manag*” OR “self-management program*” OR “health promotion” OR “disease education” OR “chronic disease education” OR “education* program*” OR “education* resource*” OR “patient education”) OR

AB (“health education” OR “community education” OR “community education program*” OR “community health” OR “community health program*” OR “self-manag*” OR “self-management program*” OR “health promotion” OR “disease education” OR “chronic disease education” OR “education* program*” OR “education* resource*” OR “patient education”)

)

AND

(

TI("impact" OR "outcome" OR "effect" OR "evaluation" OR "intervention effect*" OR "program evaluation" OR "effectiveness” OR "feasibility" OR "acceptability" OR "usability" OR "implementation" OR "adoption" OR "barrier" OR "facilitator" OR "knowledge" OR "health knowledge" OR "awareness" OR "understanding" OR "behavior change" OR "behaviour change" OR "self-efficacy" OR "health behavior" OR "attitude change" OR "adherence" OR “delivery”) OR

AB ("impact" OR "outcome" OR "effect" OR "evaluation" OR "intervention effect*" OR "program evaluation" OR "effectiveness” OR "feasibility" OR "acceptability" OR "usability" OR "implementation" OR "adoption" OR "barrier" OR "facilitator" OR "knowledge" OR "health knowledge" OR "awareness" OR "understanding" OR "behavior change" OR "behaviour change" OR "self-efficacy" OR "health behavior" OR "attitude change" OR "adherence" OR “delivery”))

**Limiters** - Publication Date: 20150101-20251231; Age Groups: Child: 6-12 years

**ERIC - 882 results returned 30/4/25**

(child* OR pediatric* OR paediatric*)

AND

("chronic pain*" OR "widespread chronic pain*" OR "chronic primary pain*" OR "chronic secondary pain*" OR "persistent pain*" OR "ongoing pain" OR "long-term pain*" OR "chronic disease" OR "chronic condition*" OR "chronic illness" OR "long-term illness" OR "disability" OR "Cancer" OR "Diabetes" OR "Autism" OR "cerebral palsy" OR "intellectual disability*" OR "Attention deficit hyperactivity disorder" OR "brain injury" OR "mental health" OR "irritable bowel disease")

AND

("health education" OR "community education" OR "community education program*" OR "community health" OR "community health program*" OR "self-manag*" OR "self-management program*" OR "health promotion" OR "disease education" OR "chronic disease education" OR "education* program*" OR "education* resource*" OR "patient education" OR "health literacy")

AND

("impact" OR "evaluation" OR "program evaluation" OR "effectiveness" OR "feasibility" OR "acceptability" OR "usability" OR "implementation" OR "adoption" OR "barrier" OR "facilitator" OR "knowledge" OR "health knowledge" OR "behavior change" OR "behaviour change" OR "self-efficacy" OR "health behavior" OR "attitude change" OR "adherence" OR "delivery")

Limited by:

Date: From 2015 to 2025

**SCOPUS - 6406 results 30/4/25**

( TITLE-ABS-KEY ( "chronic pain*" OR "widespread chronic pain*" OR "chronic primary pain*" OR "chronic secondary pain*" OR "persistent pain*" OR "ongoing pain" OR "long-term pain*" OR "chronic disease" OR "chronic condition*" OR "chronic illness" OR "long-term illness" OR "disability" OR "Cancer" OR "Diabetes" OR "Autism" OR "cerebral palsy" OR "intellectual disability*" OR "Attention deficit hyperactivity disorder" OR "brain injury" OR "mental health" OR "idiopathic arthritis" OR "irritable bowel disease" ) AND TITLE-ABS-KEY ( child* OR pediatric* OR paediatric* ) AND TITLE-ABS-KEY ( "health education" OR "community education" OR "community education program*" OR "community health" OR "community health program*" OR "self-manag*" OR "self-management program*" OR "health promotion" OR "disease education" OR "chronic disease education" OR "education* program*" OR "education* resource*" OR "patient education" ) AND TITLE-ABS-KEY ( "impact" OR "outcome" OR "effect" OR "evaluation" OR "intervention effect*" OR "program evaluation" OR "effectiveness" OR "feasibility" OR "acceptability" OR "usability" OR "implementation" OR "adoption" OR "barrier" OR "facilitator" OR "knowledge" OR "health knowledge" OR "awareness" OR "understanding" OR "behavior change" OR "behaviour change" OR "self-efficacy" OR "health behavior" OR "attitude change" OR "adherence" OR "delivery" ) ) AND PUBYEAR > 2014 AND PUBYEAR < 2026 AND ( LIMIT-TO ( EXACTKEYWORD , "Child" ) )

**COCHRANE LIBRARY - included both trials and reviews - 1867 results returned 30/4/25**

ID

#1 (child* OR pediatric* OR paediatric*):ti,ab,kw

#2 MeSH descriptor: [Child] explode all trees

#3 MeSH descriptor: [Chronic Pain] explode all trees

#4 MeSH descriptor: [Chronic Disease] explode all trees

#5 MeSH descriptor: [Health Education] explode all trees

#6 MeSH descriptor: [Self-Management] explode all trees

#7 MeSH descriptor: [Health Promotion] explode all trees

#8 MeSH descriptor: [Patient Education as Topic] explode all trees

#9 ((chronic NEXT pain* OR widespread NEXT chronic NEXT pain* OR chronic NEXT primary NEXT pain* OR chronic NEXT secondary NEXT pain* OR persistent NEXT pain* OR ongoing NEXT pain OR long-term NEXT pain* OR chronic NEXT disease OR chronic NEXT condition* OR chronic NEXT illness OR long-term NEXT illness OR disability OR Cancer OR Diabetes OR Autism OR cerebral NEXT palsy OR intellectual NEXT disability* OR Attention NEXT deficit NEXT hyperactivity NEXT disorder OR brain NEXT injury OR mental NEXT health OR idiopathic NEXT arthritis OR irritable NEXT bowel NEXT disease)):ti,ab,kw

#10 #1 OR #2

#11 #3 OR #4 OR #9

#12 (((health NEXT education OR community NEXT education OR community NEXT education NEXT program* OR community NEXT health OR community NEXT health NEXT program* OR self-manag* OR self-management NEXT program* OR health NEXT promotion OR disease NEXT education OR chronic NEXT disease NEXT education OR education* NEXT program* OR education* NEXT resource* OR patient NEXT education))):ti,ab,kw

#13 ((impact OR outcome OR effect OR evaluation OR intervention NEXT effect* OR program NEXT evaluation OR effectiveness OR feasibility OR acceptability OR usability OR implementation OR adoption OR barrier OR facilitator OR knowledge OR health NEXT knowledge OR awareness OR understanding OR behavior NEXT change OR behaviour NEXT change OR self-efficacy OR health NEXT behavior OR attitude NEXT change OR adherence OR delivery)):ti,ab,kw

#14 #5 OR #6 OR #7 OR #8 OR #12

#15 #10 AND #11 AND #14 AND #13 with Cochrane Library publication date Between Jan 2015 and Dec 2025

**PSYCINFO - 391 results returned 30/4/25**

(pediatrics.sh. or exp child/ or (pe$diatric* or child* or youth*).ti,ab.) and (chronic pain.sh. or exp chronic disease/ or (chronic pain* or widespread chronic pain* or chronic primary pain* or chronic secondary pain* or persistent pain* or ongoing pain or long-term pain* or musculoskeletal pain or chronic disease or chronic condition* or chronic illness or long-term illness or disability or Cancer or Diabetes or Autism or cerebral palsy or intellectual disability* or Attention deficit hyperactivity disorder or brain injury or mental health or idiopathic arthritis or irritable bowel disease).ti,ab.) and ((health education or self-management or health promotion or patient education as topic).sh. or (health education or community education or community education program* or community health or community health program* or self-manag* or self-management program* or education* program* or education* resource* or patient education or health literacy).ti,ab.) and (health knowledge, attitudes, practice/ or "treatment adherence and compliance"/ or health education.sh. or program evaluation.sh. or exp program evaluation/ or exp feasibility studies/ or (impact or evaluation or program evaluation or effective or feasibility or acceptability or usability or implementation or adoption or knowledge* or health knowledge or behaviour change or self-efficacy or adherence or delivery).ti,ab.)

Limit to 2015-2025

Limit to child (6-12 years)

From PsycInfo 28/11/25:

| 1. | (pediatrics.sh. or exp child/ or (pe$diatric* or child* or youth*).ti,ab.) and (chronic pain.sh. or exp chronic disease/ or (chronic pain* or widespread chronic pain* or chronic primary pain* or chronic secondary pain* or persistent pain* or ongoing pain or long-term pain* or musculoskeletal pain or chronic disease or chronic condition* or chronic illness or long-term illness or disability or Cancer or Diabetes or Autism or cerebral palsy or intellectual disability* or Attention deficit hyperactivity disorder or brain injury or mental health or idiopathic arthritis or irritable bowel disease).ti,ab.) and ((health education or self-management or health promotion or patient education as topic).sh. or (health education or community education or community education program* or community health or community health program* or self-manag* or self-management program* or education* program* or education* resource* or patient education or health literacy).ti,ab.) and (health knowledge, attitudes, practice/ or "treatment adherence and compliance"/ or health education.sh. or program evaluation.sh. or exp program evaluation/ or exp feasibility studies/ or (impact or evaluation or program evaluation or effective or feasibility or acceptability or usability or implementation or adoption or knowledge* or health knowledge or behaviour change or self-efficacy or adherence or delivery).ti,ab.) |
| --- | --- |
| 2. | limit 1 to (yr="2015 - 2025" and "180 school age (age 6 to 12 yrs)") |
